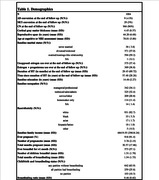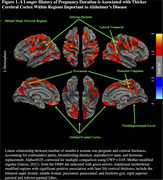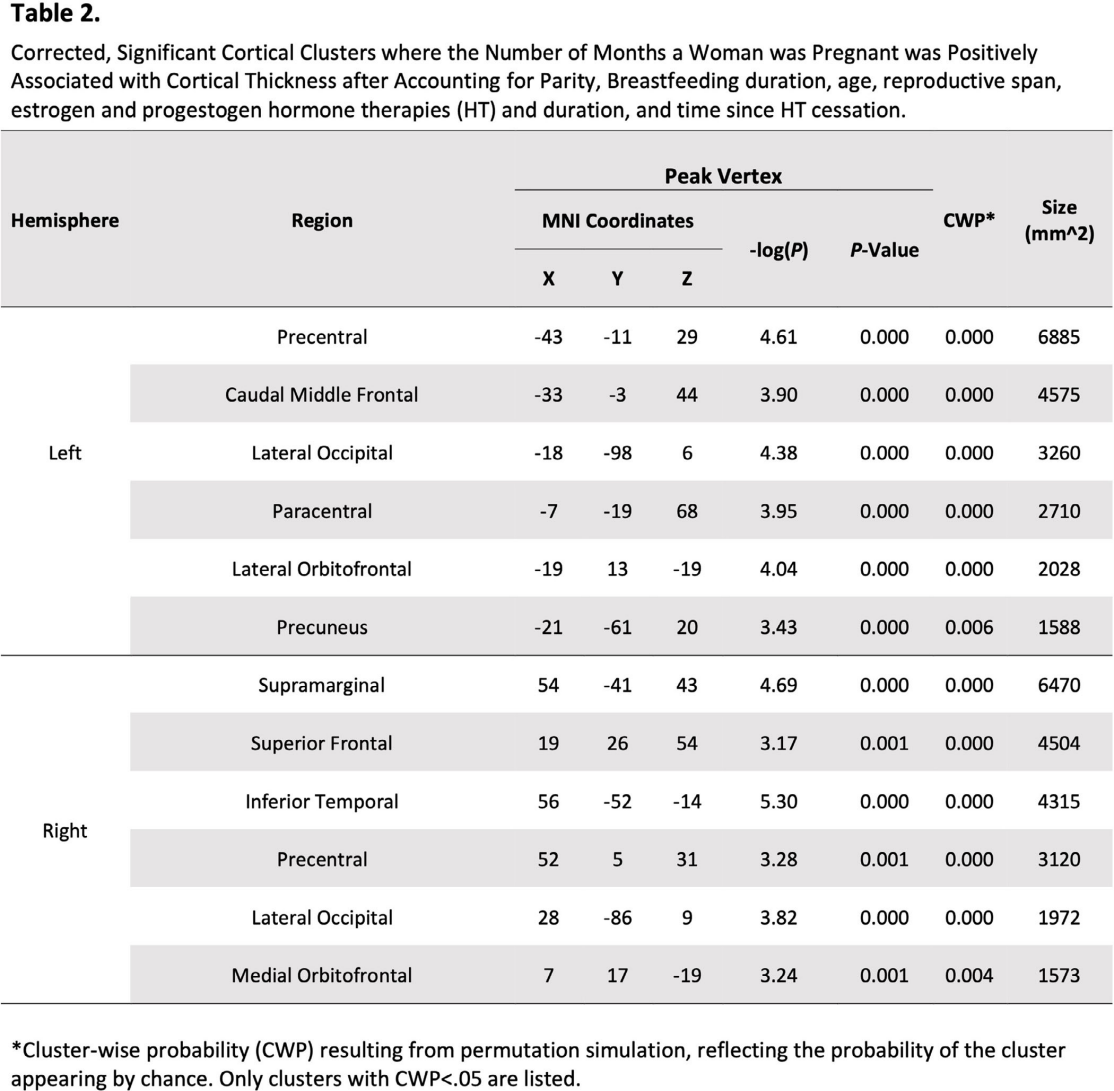# More Cumulative Time Spent Pregnant is Associated with Thicker Cerebral Cortex in Postmenopausal Women

**DOI:** 10.1002/alz.092707

**Published:** 2025-01-09

**Authors:** Jennifer E. Bramen, Emily S. Popa, Dayoon Kwon, Marcus Chang, Sonel Raj, Carolyn Crandall, Mark A. Espeland, Verna R. Porter, Prabha Siddarth, Molly Fox

**Affiliations:** ^1^ Saint John’s Cancer Institute at Providence Saint John’s Health Center, Santa Monica, CA USA; ^2^ Pacific Brain Health Center, Pacific Neuroscience Institute and Foundation, Santa Monica, CA USA; ^3^ UCLA Fielding School of Public Health, Los Angeles, CA USA; ^4^ UCLA, Los Angeles, CA USA; ^5^ UCLA David Geffen School of Medicine, Los Angeles, CA USA; ^6^ Wake Forrest School of Medicine, Winston‐Salem, NC USA; ^7^ Pacific Brain Health Center, Pacific Neuroscience Institute Foundation, Santa Monica, CA USA; ^8^ Providence Saint John’s Health Center, Santa Monica, CA USA; ^9^ David Geffen School of Medicine at University of California Los Angeles, Los Angeles, CA USA

## Abstract

**Background:**

Matrescence, like adolescence, is a critical period for neurodevelopment characterized by hormonal changes that reshape the brain in preparation for new experiences and subsequent learning. Women exhibit greater age‐matched Alzheimer’s disease (AD) risk than men, yet little is known about long‐term neurological health consequences of reproduction (Buckley, 2019), the defining biological difference between the sexes. We tested the hypothesis that greater number of months pregnant would be positively associated with cortical thickness (CT), particularly in regions within the default mode network (DMN). DMN disruption is well‐established in AD pathology (Dennis, 2014). Research also indicates that synaptic pruning within the DMN during pregnancy is related to improved maternal attachment and reduced hostility toward the infant, with these changes persisting post‐partum (Hoekzema, 2017; Garcia, 2021). Moreover, in late life, beneficial changes due to motherhood have been shown in the DMN (Orchard, 2021).

**Method:**

We used data from 1004 older women from the Women’s Health Initiative Magnetic Resonance Imaging (MRI) study (Coker, 2009) (**Table 1**). CT was estimated from T1‐weighted MRI using FreeSurfer. The effect of cumulative pregnancy duration on mean CT was assessed using a linear mixed model, controlling for parity (number of complete pregnancies), breastfeeding duration, age, reproductive span, estrogen, and progestogen hormone therapies (HT) and duration, and time since HT cessation. Cortical surface analyses used linear regression, accounting for the same confounders, and were corrected for multiple comparisons using cluster‐wise probability.

**Results:**

The number of months a woman was pregnant was positively associated with global cortical thickness (b = 0.002, p = 0.002). Cortical surface analysis revealed only positive regional associations with CT, including most regions of the DMN (**Figure 1; Table 2**), as well as several clusters outside the DMN. Notably, the anterior cingulate (ACG) did not show a significant association.

**Conclusions:**

This work supports the hypothesis that pregnancy may be beneficial for late‐life brain health, particularly in regions important to AD‐pathology such as the DMN, with benefits extending beyond the effect of parity and breastfeeding. Future directions of this work include subcortical analysis and examining whether genetic risk for AD (such as APOE) modifies the relationship between pregnancy and cortical atrophy.